# Interplay between ceRNA and Epigenetic Control of microRNA: Modelling Approaches with Application to the Role of Estrogen in Ovarian Cancer

**DOI:** 10.3390/ijms23042277

**Published:** 2022-02-18

**Authors:** Tzy-Wei Huang, Frank H. C. Cheng, Ching-Cher Sanders Yan, Yu-Ming Chuang, Chien-Hong Cho, Hung-Cheng Lai, Shih-Feng Shieh, Michael W. Y. Chan, Je-Chiang Tsai

**Affiliations:** 1 Department of Mathematics, National Chung Cheng University, Min-Hsiung, Chia-Yi 621, Taiwan; twhwang0637@gmail.com; 2 Department of Biomedical Sciences, National Chung Cheng University, Min-Hsiung, Chia-Yi 621, Taiwan; frcheng@iu.edu (F.H.C.C.); ccu403250029@gmail.com (Y.-M.C.); 3 Epigenomics and Human Disease Research Center, National Chung Cheng University, Min-Hsiung, Chia-Yi 621, Taiwan; 4 Indiana University School of Medicine-Bloomington, Indiana University, Bloomington, IN 47405, USA; 5 Institute of Chemistry, Academia Sinica, Nangang District, Taipei 115, Taiwan; ccsyan@gmail.com; 6 Department of Applied Mathematics, National Sun Yat-Sen University, Gushan District, Kaohsiung 804, Taiwan; chcho@math.nsysu.edu.tw; 7 Department of Obstetrics and Gynecology, Shuang Ho Hospital, Taipei Medical University, Zhonghe District, Taipei 235, Taiwan; hclai@s.tmu.edu.tw; 8 Department of Obstetrics and Gynecology, Tri-Service General Hospital, National Defense Medical Center, Neihu District, Taipei 114, Taiwan; 9 Department of Mathematics, National Taiwan Normal University, Wenshan District, Taipei 106, Taiwan; sfshieh@ntnu.edu.tw; 10 Department of Mathematics, National Tsing Hua University, East District, Hsinchu 300, Taiwan; 11 Brain Research Center, National Tsing Hua University, East District, Hsinchu 300, Taiwan; 12 National Center for Theoretical Science, National Taiwan University, Da’an District, Taipei 106, Taiwan

**Keywords:** deterministic model, epigenetics, microRNA, target-translated protein, ceRNA, ovarian cancer

## Abstract

MicroRNAs (miRNAs) play an important role in gene regulation by degradation or translational inhibition of the targeted mRNAs. It has been experimentally shown that the way miRNAs interact with their targets can be used to explain the indirect interactions among their targets, i.e., competing endogenous RNA (ceRNA). However, whether the protein translated from the targeted mRNAs can play any role in this ceRNA network has not been explored. Here we propose a deterministic model to demonstrate that in a network of one miRNA interacting with multiple-targeted mRNAs, the competition between miRNA-targeted mRNAs is not sufficient for the significant change of those targeted mRNA levels, while dramatic changes of these miRNA-targeted mRNAs require transcriptional inhibition of miRNA by its target proteins. When applied to estrogen receptor signaling pathways, the miR-193a targets E2F6 (a target of estrogen receptor), c-KIT (a marker for cancer stemness), and PBX1 (a transcriptional activator for immunosuppressive cytokine, IL-10) in ovarian cancer, such that epigenetic silencing of miR-193a by E2F6 protein is required for the significant change of c-KIT and PBX1 mRNA level for cancer stemness and immunoevasion, respectively, in ovarian cancer carcinogenesis

## 1. Introduction

Epigenetic alterations, including DNA methylation, histone modifications, and non-coding RNA expression, play an important role in gene regulation, yet aberrant epigenetic modifications are now considered as a hallmark of cancer [1,2,3,4]. Epigenetic silencing of a tumor suppressor gene at the promoter region by DNA methylation and histone modifications has been widely described [5,6]. Among them, the enzymatic component of the PRC2 complex, EZH2, a histone methyltransferase for H3K27, can recruit DNA methyltransferase (DNMT) to the DNA, thus linking DNA methylation and histone modification together in mediating transcriptional repression [7]. On the other hand, microRNA (miR), which is either generated from a miR gene or part of an intron, can bind to the targeted mRNAs to inhibit translation or promote mRNA degradation, depending on the location of the miR response element (MRE) [8]. Previous studies by Pandolfi et al. demonstrated that expression of the miR-targeted mRNAs (containing similar MRE) can compete for the binding of the same miRs, thus affecting the expression of another miR-targeted mRNAs [9,10]. This competing endogenous RNA (ceRNA) phenomenon has been shown to play an important role in cancer development [9]. However, the interplay between DNA methylation, histone modifications, and ceRNA are not fully explored.

In this study, we describe a mathematical model, using ovarian cancer as an example, to demonstrate how DNA methylation and histone modification participate in ceRNA phenomenon, and eventually affect ovarian cancer stemness and cancer immunoevasion.

## 2. Results

**Figure 1:**
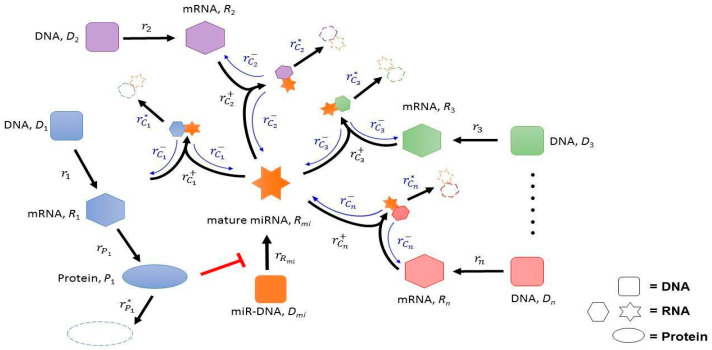
A qualitative network involving $R_{mi}$ and its target mRNAs. (Arrow) Activation or upregulation. (Hammerheads) Inhibition or downregulation. Table 1 gives the notations of genes, complexes, and rate functions. The descriptions and the references for the interactions in this figure are illustrated in Table S1 of Supplementary Text S2.

Let us consider a network consisting of a miRNA, say $R_{mi}$, and n targeted mRNAs, say $R_{1}$, $R_{2}$, …, $R_{n}$. We note that $R_{mi}$ regulates the expression of its target mRNAs through $R_{mi}$ binding to mRNA targets, which in turn forms the complex $C_{i}$. This complex will then be degraded, resulting in the target mRNA degradation. We also hypothesize the transcriptional inhibition of $R_{mi}$ by targeted mRNA $R_{1}$-mediated epigenetic silencing. We assume that free $R_{mi}$ and its target mRNA $R_{i}$ form a complex $C_{i}$ with association rate function $r_{C_{i}}^{+}$ and dissociation rate function $r_{C_{i}}^{-}$. Then, the mutual interaction between $R_{mi}$ and its target mRNA $R_{i}$’s is generated in the network system, as depicted in Figure 1.

### 2.1. Transcriptional Inhibition by Protein $P_{1}$ Is Necessary for a Dramatic Change of $R_{i}$’s

The ceRNA mechanism of $R_{mi}$ indicates that an upregulation of its targeted mRNA $R_{1}$ will sponge $R_{mi}$ to the MRE of this mRNA. Such upregulation will in turn promote the level of the other targeted mRNAs due to less $R_{mi}$ binding to those mRNAs. On the other hand, the ceRNA mechanism alone may not be able to allow target mRNAs to have a significant change as the transcriptional rate of $R_{1}$ is increased. Indeed, under the assumption on the qualitative dependence of rate functions on $R_{mi}$, its targeted mRNAs, and their complex (as indicated in Table 2 of the Materials and Methods section), we can mathematically show that transcriptional inhibition of $R_{mi}$ by protein $P_{1}$ (red inhibitory line in Figure 1) is necessary for the network system to possess a SN bifurcation (see Supplementary Text S1), and thus necessary for carcinogenesis.

### 2.2. Inhibition of miR-193a by E2F6-Mediated Epigenetic Silencing in Ovarian Cancer

**Figure 2:**
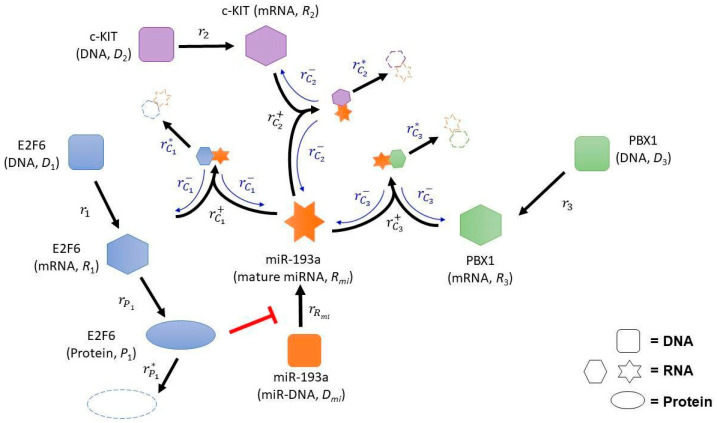
A qualitative network involving miR-193a and its target mRNAs. (Arrow) Activation or upregulation. (Hammerheads) Inhibition or downregulation. Description of each reaction is given in Table S1 (from the file: Supplementary Text S2).

Ovarian cancer is the most common gynecological cancer worldwide [11]. Although several studies have demonstrated that estrogen has been involved in the development of ovarian carcinogenesis [12,13,14], anti-estrogen therapy is only partially effective in the treatment of ovarian cancer [13,15]. Understanding the “missing-link” behind this inconsistency may help in better understanding the cause of ovarian cancer, and thus the development of a better targeted therapy for this deadly disease [1,2,3,4]. In this regard, we have previously demonstrated that estrogen may be linked to ovarian cancer through a microRNA, miR-193a [16,17,18], which has been found to be a tumor suppressor microRNA in several human cancers such as lung, pancreatic, oral, and colon cancer [19,20,21,22]. We therefore determined to use miR-193a as an example. The network scheme of ovarian carcinogenesis corresponding to that in Figure 1 is depicted in Figure 2.

Computational analysis using several miRNA databases found that one of the miR-193a targets is E2F6, which is also an estrogen receptor (ER) target [23]. As different from other E2F families, E2F6 is a transcriptional repressor by recruiting DNMT and EZH2 [24]. In this regard, the presence of E2F6 binding at the promoter region of miR-193a suggests that miR-193a can be suppressed by E2F6-mediated epigenetic silencing. Furthermore, biological experiments also found that miR-193a can target mRNAs, such as c-KIT [16,17] and PBX1 (data not shown), as depicted in Figure 2.

Indeed, the aforementioned mathematical result is not confined to the network of miR-193a and those three targeted mRNAs, but can be extended to the network of all competing endogenous networks. Specifically, for the general case that there are multiple miR-193a targeted mRNAs, mathematical analysis still indicates (see Supplementary Text S1) that the transcriptional inhibition of miR-193a by E2F6 protein is necessary for the rapid increase in c-KIT mRNA level.

### 2.3. Missing-Link in the Inconsistency of the Role of Estrogen

As mentioned previously, clinical evidence indicates that anti-estrogen therapy is only partially effective in the treatment of ovarian cancer [13,15]. We will exploit system (1), which is associated with the network scheme in Figure 1 and defined in the Matherials and Methods section, to derive two possible scenarios to give a partial answer to this puzzle. To observe this, we need to specify the rate functions on the right-hand side of system (1). To be precise, let the transcriptional rate function of E2F6 mRNA $r_{1}=k_{1}R_{1}.$ Here, the transcriptional rate parameter $k_{1}$ depends on the level of hormonal stimulation, and thus can be viewed as an experimentally controllable parameter associated with the addition of hormones. Next, we choose the following kinetic form for miRNA $R_{mi}$ transcription:

$$r_{Rmi}=\frac{k_{Rmi}}{1+KP_{1}}.$$

Here, $k_{Rmi}$ is the transcriptional rate of miR-193a. The fact that EZH2 can be recruited to the miR-193a promoter only when E2F6 protein is bound to the miR-193a promoter enables one to view K as a measure of the combined strength of the miR-193a transcriptional inhibition by E2F6 protein and the epigenetic silencing of miR-193a, via EZH2 and DNMT3b. The other rate functions and their associated rate parameters are stated in Supplementary Text S2 of the Supporting Materials.

**Figure 3:**
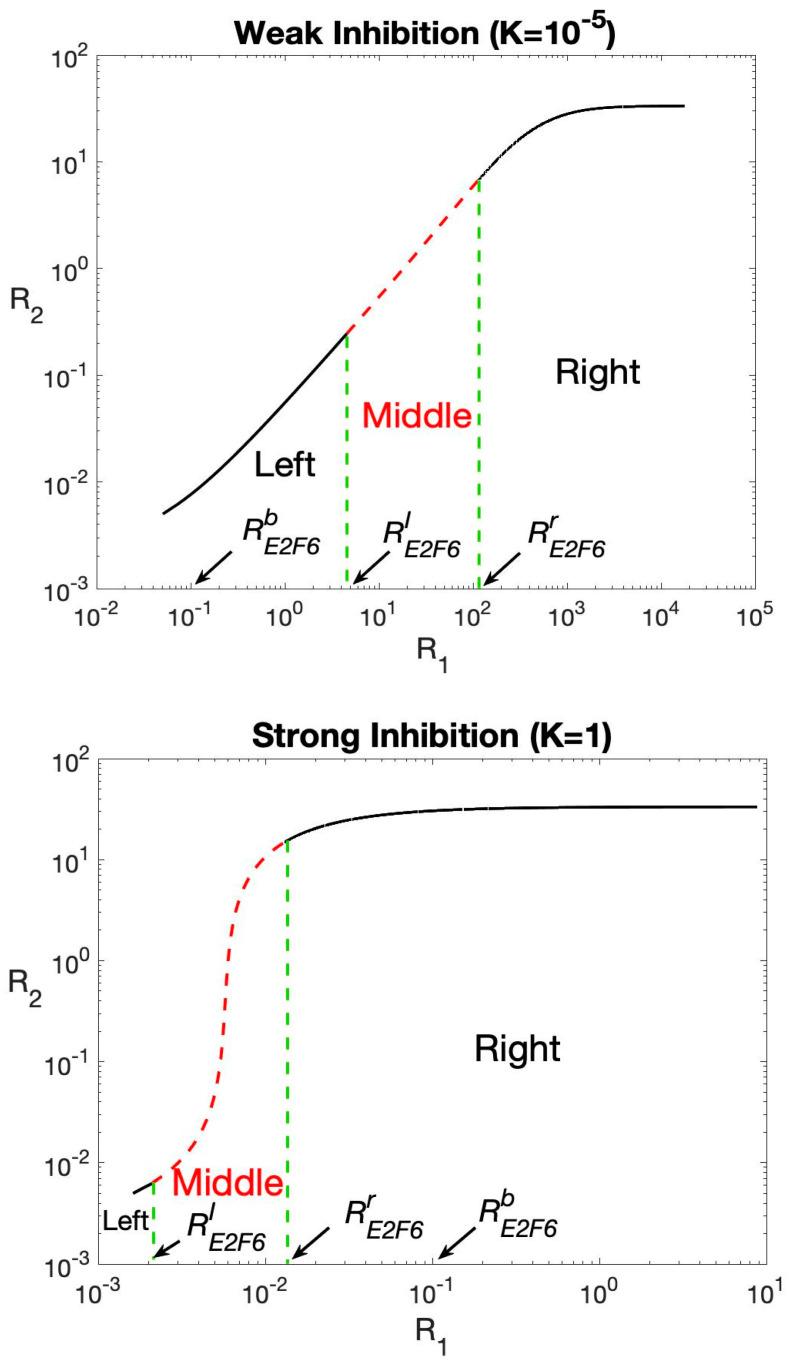
The relation curve Γ between the levels of variables $R_{1}(=R_{E2F6})$ and $R_{2}(=R_{c-kit})$ of steady states as the transcriptional rate parameter $k_{1}$ of E2F6 is varied. The top panel is for the case of the weak inhibition strength of E2F6 on miR-193a (small K ), while the bottom panel is for the strong inhibition strength of E2F6 on miR-193a (large K ). The red dashed lines correspond to unstable steady states, while the green dashed lines mark the relevant points. The parameter values are given in Supplementary Text S2.

Now, we specify the relation between the E2F6 mRNA and other miR-193a targeted mRNAs levels of steady states of system (1). Indeed, as the E2F6 transcription rate $k_{1}$ is varied from the low value to the high value, the relationship between the E2F6 mRNA level $R_{1}$ and miR-193a targeted mRNA level $R_{2}$ of steady states traces out an increasing curve, say Γ, as depicted in Figure 3. The curve Γ consists of three parts: the leftmost branch, the middle branch (associated with the red dashed lines in Figure 3), and the rightmost branch. The leftmost and rightmost branches correspond to stable steady states, while the middle branch corresponds to unstable steady states. Furthermore, note that the c-KIT level of a steady state associated with the rightmost branch of Γ is 10 to 1000-fold greater compared with that on the leftmost branch. This observation suggests that the states on the rightmost branch of Γ are associated with the carcinogenesis overexpression of c-KIT mRNA, while those on the leftmost branch are associated with the normal c-KIT mRNA level. 

To facilitate the discussion, denote by $R_{E2F6}^{l}$ (respectively, $R_{E2F6}^{r}$), the critical E2F6 mRNA level associated with the left (respectively, right) end point of the middle branch of Γ. Here, the superscript of $R_{E2F6}^{l}$ (respectively, $R_{E2F6}^{r}$) means left (respectively, right). As the transcription rate parameter $k_{1}$ is increased from the low value to the high value, the steady state will first move along the leftmost branch and the corresponding c-KIT level gradually increases, then, due to the instability of steady states on the middle branch, the steady state will be switched to the rightmost branch at the critical value $R_{E2F6}^{r}$ and move along the rightmost branch for $R_{1}$* > *$R_{E2F6}^{r}$, which thus turns on the carcinogenesis overexpression of c-KIT mRNA. On the other hand, if the steady state is initially on the rightmost branch with $R_{1}$* > *$R_{E2F6}^{r}$, then, due to the stable feature of steady states on the upper branch, it follows that as the transcriptional rate parameter $k_{1}$ is decreased from the high value to the low value, the steady state will first move along the rightmost branch and the corresponding c-KIT level gradually decreases, and then, due to the instability of steady states on the middle branch, the steady state will be descended to the leftmost branch at the critical value $R_{E2F6}^{l}$ provided that $R_{E2F6}^{l}$ is larger than the basal level $R_{E2F6}^{b}$ of E2F6 mRNA, and then move along the leftmost branch for $R_{1}$* < *$R_{E2F6}^{l}$, which thus recovers the normal level of c-KIT mRNA. 

However, if $R_{E2F6}^{l}$ is less than the basal level $R_{E2F6}^{b}$, then the steady state will remain on the rightmost branch, not descend to the lower branch, which thus cannot switch off the carcinogenesis overexpression of c-KIT mRNA. The former case corresponds to the weak inhibition strength of E2F6 on miR-193a (small K) as illustrated in the top panel of Figure 3, while the latter case corresponds to the strong inhibition strength of E2F6 on miR-193a (large K) as illustrated in the bottom panel of Figure 3.

To summarize, for the cells with lower E2F6 inhibition efficiency, an increase of E2F6 transcription rate from low value to high value can promote the expression of c-KIT mRNA, and, conversely, a retraction of E2F6 transcription rate can recover the normal c-KIT mRNA level. In contrast, for the cell with higher E2F6 inhibition efficiency, an increase of E2F6 transcription rate up through the threshold transcriptional rate will trigger the overexpression of c-KIT mRNA. However, a retraction of E2F6 transcription rate cannot switch off the overexpression of c-KIT mRNA. Thus, the reversible/irreversible feature of c-KIT mRNA regulation processes by different E2F6 inhibition strength can partially explain the clinical observation that anti-estrogen therapy is only partially effective in the treatment of ovarian cancer.

### 2.4. Contribution of E2F6 mRNA in Anti-Tumor Immune Response 

On the other hand, suppression of anti-cancer immunity may be an important mechanism for ovarian carcinogenesis. Such anti-tumor immunity may be attenuated by cancer cells expressing immunosuppressive cytokines, such as IL-10 [25]. It is interesting to note that miR-193a provides a possible link between cancer stemness and immunoevasion [26], as PBX1 (which is another predicted target of miR-193a), as well as the transcriptional activator for the immunosuppressive cytokine IL-10. In this regard, upregulation of PBX1 can result in the transcription of IL-10 and corresponding immunosuppression. The regulation process of miR-193a targeted mRNA (c-KIT mRNA) by E2F6, as demonstrated in the aforementioned result, can also be observed in PBX1. 

## 3. Discussion

In this paper, we proposed a deterministic model to demonstrate that in a network of one miRNA interacting with multiple targeted mRNAs, the ceRNA mechanism alone is not able to allow target mRNAs to have a significant change as the transcriptional rate of one target mRNA is increased. In this regard, transcriptional inhibition of miRNA by its target protein can induce dramatic changes of these miRNA-targeted mRNAs. Therefore, miRNA-targeted mRNAs’ behavior is the competition between the ceRNA mechanism and epigenetic silencing of miRNA by its target protein.

When applied to estrogen receptor signaling pathways, our mathematical result suggests that the inhibition of miR-193a by E2F6 protein, which recruits EZH2 and DNMT3b, is required for the overexpression of c-KIT and PBX1 mRNAs. The regulation of expression of miR-193a targeted mRNAs is the result of the competition between ceRNA mechanism among those miR-193a targets and promoter hypermethylation of miR-193a. To be specific, for cells with low inhibition efficiency of E2F6, the inhibition of miR-193a expression by E2F6 protein is not strong enough to induce promoter methylation of miR-193a. Hence, an increase in E2F6 expression will result in a gradual change of all other target mRNAs of miR-193a, through a ceRNA manner. Conversely, a reduction of E2F6 expression may reduce c-KIT and PBX1 expression. Thus, for low inhibition efficiency of E2F6, the regulation process of c-KIT and PBX1 expression is reversible.

On the other hand, for cells with high inhibition efficiency of E2F6, our model predicts a threshold expression level ($R_{E2F6}^{l}$) of E2F6 which determines the dominant role in the regulation of c-KIT and PBX1 expression. Indeed, for the expression level of E2F6 below $R_{E2F6}^{l}$, the inhibition of miR-193a expression by E2F6 protein is not strong enough. So, the ceRNA mechanism dominates the regulation of c-KIT and PBX1 expression, and thus they will change gradually as E2F6 expression is increased. However, as the expression level of E2F6 exceeded $R_{E2F6}^{l}$, the strength of inhibition of miR-193a by E2F6 protein is large enough to induce DNA methylation of miR-193a, and thus fewer free miR-193a can bind to its target mRNAs, which in turn leads to the significant increase of c-KIT and PBX1 expression. Further, due to the epigenetic silencing of miR-193a through DNA methylation and high inhibition efficiency of E2F6 protein, a downregulation of E2F6 expression cross the threshold level $R_{E2F6}^{l}$ is not able to recover the normal level of c-KIT and PBX1 expression. 

Consistent with this mathematical model, a recent study also demonstrated that inhibition of E2F6 suppressed tumor growth and migration in ovarian cancer [27]. Furthermore, our result also explained why anti-estrogen therapy is only partially effective in ovarian cancer, despite the fact that estrogen receptors are overexpressed in several sub-types of ovarian cancer [28]. Indeed, epigenetic therapy targeting EZH2 can not only suppress cancer stemness [29] but also restore anti-tumor immune response in ovarian cancer [30].

This study has several limitations: although we have recently verified the role of the E2F6 ceRNA network in the epigenetic control of miR-193a [18], the role of such a network in the regulation of PBX1 and the subsequent anti-tumor immune response requires experimental validation. Secondly, the total effect of the miR-193a transcriptional inhibition by E2F6 protein and the epigenetic silencing of miR-193a, via EZH2 and DNMT3b, is incorporated into the rate function $r_{R_{mi}}$ for miRNA $R_{mi}$ transcription. This description seems to be oversimplified. A more detailed modelling analysis on this transcriptional inhibition warrants further investigation. 

## 4. Materials and Methods

### 4.1. Outline of the Strategy

**Figure 4:**
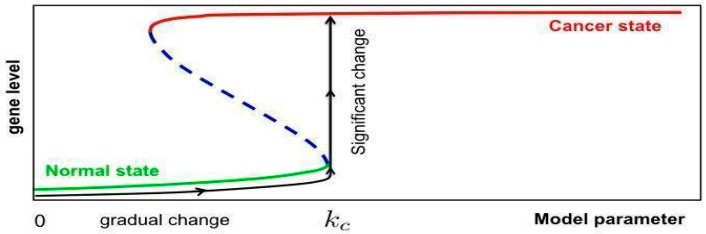
A generic illustration of a SN bifurcation modelling the switch between normal and cancer states.

It is known that the significant change in any particular mRNAs is predisposed to human diseases/cancer. Mathematically, this can be modelled as the onset of a saddle-node (SN) bifurcation in the associated reaction network, which means that a particular gene is switched from one state (normal state) associated with low level to another state (cancer state) associated with high level, as illustrated in Figure 4.

### 4.2. The Model 

The kinetic equations for the reaction scheme depicted in Figure 1 are given by the following system of differential equations:

(1) $$\begin{array}{c} \frac{dR_{mi}}{dt}=r_{R_{mi}}-r_{C_{1}}^{+}-r_{C_{2}}^{+}-\cdots -r_{C_{N}}^{+}+r_{C_{1}}^{-}+r_{C_{2}}^{-}+\cdots +r_{C_{N}}^{-}—r_{R_{mi}}^{*},\\ \;\;\frac{dP_{1}}{dt}=r_{P_{1}}-r_{P_{1}}^{*},\\ \;\;\frac{dR_{i}}{dt}=r_{i}-r_{C_{i}}^{+}+r_{C_{i}}^{-}-r_{i}^{*},\\ \;\;\frac{dC_{i}}{dt}=r_{C_{i}}^{+}-r_{C_{i}}^{-}-r_{C_{i}}^{*},\;i=1,2,\cdots ,n, \end{array}$$

where $\frac{dR_{mi}}{dt}$ represents the rate of change of $R_{mi}$ with respect to time t (i.e., differentiation with respect to time t), the other terms on the left-hand side of (1) are similarly defined, and the other state variables and the rate functions on the right-hand side of (1) are defined in the Table 1.

### 4.3. Main Model Assumption

Now we state the main assumption about the model. Indeed, we hypothesize that the transcriptional inhibition of $R_{mi}$ by its targeted protein $P_{1}$-mediated epigenetic silencing through DNA methylation and histone modification [1]. Therefore, the transcriptional rate function $r_{R_{mi}}$ of $R_{mi}$ is decreased as the level of $P_{1}$ is increased. As we will see, this hypothesis is crucial for the significant change of mRNAs $R_{i}$’s as the transcriptional rate of mRNA $R_{1}$ is increased.

### 4.4. Model Rate Functions 

In principle, one cannot determine the specific form of rate functions. However, without any stimuli, it is commonly accepted that the transcription rates of mRNAs are constants (i.e., the $r_{i}$’sare constants), that the degradation rate function (e.g., $r_{R_{mi}}^{*}$) linearly depends on the level of gene or complex (e.g., $r_{R_{mi}}^{*}=kR_{mi}$ with k being the rate parameter), and that the translation rate functions of proteins depends on the level of its corresponding mRNA linearly. The association rate function of the complex $R_{mi}$-$R_{i}$ can be assumed to be proportional to the product of the corresponding levels of $R_{mi}$ and $R_{i}$ according to the mass-action law. We summarize the qualitative dependence of genes or complexes in Table 2, where the sign + (respectively, -) means that the rate function r (showing as a corresponding sign in the same column) increases (respectively, decreases) as the level of gene or complex (showing as a corresponding sign in the same row) increases. For instance, as $R_{mi}$ is increased, the rate function $r_{C_{i}}^{+}$ is increased, while as $P_{1}$ is increased, $r_{P_{1}}^{*}$ is increased but $r_{R_{mi}}$ is decreased. Interestingly, the qualitative monotonic dependence of rate functions in Table 2 is sufficient to deduce interesting epigenetic implications without resorting to the search of specific forms of rate functions and specific rate parameter values which would be difficult in realistic cases.

### 4.5. Steady State

Now, we attribute a steady state to system (1). To begin with, let the state vector $S=\left(R_{mi},P_{1},R_{1},\ldots ,R_{n},C_{1},\ldots ,C_{n}\right)$ and f(S) be the vector function defined by the right-hand side of system (1). Then we say that $S^{0}=\left(R_{mi}^{0},P_{1}^{0},R_{1}^{0},\ldots ,R_{n}^{0},C_{1}^{0},\ldots ,C_{n}^{0}\right)$ is a steady state of system (1) if $S^{0}$ satisfies $f\left(S^{0}\right)=0$. Thus, the steady state $S^{0}$ is a solution of system (1) which does not change in time. Roughly speaking, a steady state is a situation in which the underlying system does not undergo any change as the time evolves. Now we distinguish two types of steady states: stable and unstable. To be precise, a steady state $S^{0}$ of system (1) is said to be stable (respectively, unstable) if any solution of system (1) with small initial deviation from the steady state $S^{0}$ always returns to $S^{0}$ (respectively, moves away from $S^{0}$) as time evolves. Thus, a stable steady state of system (1) is a state which is observable in realistic situations.

Finally, we will trace the change of the steady state associated with the network system as the model parameter (such as the transcription rate parameter of mRNA $R_{1}$) is tuned. When the network system admits a SN bifurcation at some critical model parameter, the corresponding level of the other targeted mRNA $R_{i}$’s will experience a significant change as the model parameter is varied through such a critical value, thereby leading to carcinogenesis.

## 5. Conclusions

In conclusion, our mathematical model predicts the behavior of c-KIT and PBX1 through combined ceRNA mechanism and epigenetic silencing of miR-193a by E2F6. This result suggests that the inhibition of estrogen receptor signaling and E2F6 (by using DNMT or EZH2 inhibitor) may be able to suppress cancer stemness and restore anti-tumor immune response in ovarian cancer.

## Figures and Tables

**Table 1:** Notations for genes, complexes, and rate functions in system (1).

Gene/Complex	Level of Genes	Transcription Rate Translation Rate Association Rate Dissociation Rate	Degradation Rate
miRNA mRNA *R_i_* (2≤i≤n) *R_1_* – translated protein miRNA – mRNA *R_i_* complex	$R_{mi}$ $R_{i}$ $P_{1}$ $C_{i}$	$r_{R_{mi}}$ $r_{i}$ $r_{P_{1}}$ $r_{C_{i}}^{\pm }$	$r_{R_{mi}}^{*}$ $r_{i}^{*}$ $r_{P_{1}}^{*}$ $r_{C_{i}}^{*}$

**Table 2:** Dependence of rate functions on miRNA, mRNAs, and their complex.

	$r_{R_{mi}}$	$r_{P_{1}}$	$r_{C_{1}}^{+}$	$r_{C_{i}}^{+}$ (i≥2)	$r_{C_{1}}^{-}$	$r_{C_{i}}^{-}$ (i≥2)	$r_{C_{1}}^{*}$	$r_{C_{i}}^{*}$ (i≥2)	$r_{P_{1}}^{*}$
$R_{mi}$			+	+					
$P_{1}$	−								+
$R_{1}$		+	+						
$R_{i}$ (i≥2)				+					
$C_{1}$					+		+		
$C_{i}$ (i≥2)						+		+	

## Data Availability

Not applicable.

## Supplementary Materials

The following supporting information can be downloaded at: https://www.mdpi.com/article/10.3390/ijms23042277/s1.

## References

[1] Baylin S.B., Jones P.A. (2016). Epigenetic Determinants of Cancer. Cold Spring Harb Perspect. Biol..

[2] Baylin S.B., Jones P.A. (2011). A decade of exploring the cancer epigenome—Biological and translational implications. Nat. Rev. Cancer.

[3] Rodríguez-Paredes M., Esteller M. (2011). Cancer epigenetics reaches mainstream oncology. Nat. Med..

[4] Piletič K., Kunej T. (2016). MicroRNA epigenetic signatuRes. in human disease. Arch. Toxicol..

[5] Jones P.A., Issa J.P., Baylin S. (2016). Targeting the cancer epigenome for therapy. Nat. Rev. Genet..

[6] Flaus A., Downs J.A., Owen-Hughes T. (2021). Histone isoforms and the oncohistone code. Curr. Opin Genet. Dev..

[7] Wang J., Wang G.G. (2020). No Easy Way Out for EZH2: Its Pleiotropic, Noncanonical Effects on Gene Regulation and Cellular Function. Int. J. Mol. Sci..

[8] Garzon R., Marcucci G., Croce C.M. (2010). Targeting microRNAs in cancer: Rationale, strategies and challenges. Nat. Rev. Drug Discov..

[9] Poliseno L., Salmena L., Zhang J., Carver B., Haveman W.J., Pandolfi P.P. (2010). A coding-independent function of gene and pseudogene mRNAs regulates tumour biology. Nature.

[10] Salmena L., Poliseno L., Tay Y., Kats L., Pandolfi P.P. (2011). A ceRNA hypothesis: The Rosetta Stone of a hidden RNA language?. Cell.

[11] Siegel R.L., Miller K.D., Fuchs H.E., Jemal A. (2021). Cancer Statistics, 2021. CA Cancer J. Clin..

[12] Collaborative Group on Epidemiological Studies of Ovarian Cancer (2015). Menopausal hormone use and ovarian cancer risk: Individual participant meta-analysis of 52 epidemiological studies. Lancet.

[13] Beral V., Million Women Study Collaborators (2007). Ovarian cancer and hormone replacement therapy in the Million Women Study. Lancet.

[14] Hunn J., Rodriguez G.C. (2012). Ovarian cancer: Etiology, risk factors, and epidemiology. Clin. Obstet. Gynecol..

[15] Smyth J.F., Gourley C., Walker G., MacKean M.J., Stevenson A., Williams A.R., Nafussi A.A., Rye T., Rye R., Stewart M. (2007). Antiestrogen therapy is active in selected ovarian cancer cases: The use of letrozole in estrogen receptor-positive patients. Clin. Cancer Res..

[16] Cheng F.H.C., Aguda B.D., Tsai J.-C., Kochańczyk M., Lin J.M.J., Chen G.C.W., Lai H.-C., Nephew K.P., Hwang T.-W., Chan M.W.Y. (2014). A mathematical model of bimodal epigenetic control of miR-193a in ovarian cancer stem cells. PLoS ONE.

[17] Gao X.N., Lin J., Li Y.H., Gao L., Wang X.R., Wang W., Kang H.Y., Yan G.T., Wang L.L., Yu L. (2011). MicroRNA-193a represses c-kit expression and functions as a methylation-silenced tumor suppressor in acute myeloid leukemia. Oncogene.

[18] Cheng F.H.C., Lin H.Y., Hwang T.W., Chen Y.C., Huang R.L., Chang C.B., Yang W., Lin R.I., Lin C.W., Chen G.C.W. (2019). E2F6 functions as a competing endogenous RNA, and transcriptional repressor, to promote ovarian cancer stemness. Cancer Sci..

[19] Dai X., Chen X., Chen Q., Shi L., Liang H., Zhou Z., Liu Q., Pang W., Hou D., Wang C. (2015). MicroRNA-193a-3p Reduces Intestinal Inflammation in Response to Microbiota via Down-regulation of Colonic PepT1. J. Biol. Chem..

[20] Fang C., Dai C.Y., Mei Z., Jiang M.J., Gu D.N., Huang Q., Tian L. (2018). microRNA-193a stimulates pancreatic cancer cell repopulation and metastasis through modulating TGF-β_2_/TGF-βRIII signalings. J. Exp. Clin. Cancer Res..

[21] Chen D., Cabay R.J., Jin Y., Wang A., Lu Y., Shah-Khan M., Zhou X. (2013). MicroRNA Deregulations in Head and Neck Squamous Cell Carcinomas. J. Oral Maxillofac. Res..

[22] Teng Y., Ren Y., Hu X., Mu J., Samykutty A., Zhuang X., Deng Z., Kumar A., Zhang L., Merchant M.L. (2017). MVP-mediated exosomal sorting of miR-193a promotes colon cancer progression. Nat. Commun..

[23] Carroll J.S., Meyer C.A., Song J., Li W., Geistlinger T.R., Eeckhoute J., Brodsky A.S., Keeton E.K., Fertuck K.C., Hall G.F. (2006). Genome-wide analysis of estrogen receptor binding sites. Nat. Genet..

[24] Trimarchi J.M., Fairchild B., Wen J., Lees J.A. (2001). The E2F6 transcription factor is a component of the mammalian Bmi1-containing polycomb complex. Proc. Natl. Acad. Sci. USA.

[25] Dennis K.L., Blatner N.R., Gounari F., Khazaie K. (2013). Current status of interleukin-10 and regulatory T-cells in cancer. Curr. Opin. Oncol..

[26] Chung E.Y., Liu J., Homma Y., Zhang Y., Brendolan A., Saggese M., Han J., Silverstein R., Selleri L., Ma X. (2007). Interleukin-10 expression in macrophages during phagocytosis of apoptotic cells is mediated by homeodomain proteins Pbx1 and Prep-1. Immunity.

[27] An Y., Zhang J., Cheng X., Li B., Tian Y., Zhang X., Zhao F. (2020). miR-454 suppresses the proliferation and invasion of ovarian cancer by targeting E2F6. Cancer Cell Int..

[28] Paleari L., DeCensi A. (2018). Endocrine therapy in ovarian cancer: Where do we stand?. Curr. Opin. Obstet. Gynecol..

[29] Zong X., Wang W., Ozes A., Fang F., Sandusky G.E., Nephew K.P. (2020). EZH2-Mediated Downregulation of the Tumor Suppressor DAB2IP Maintains Ovarian Cancer Stem Cells. Cancer Res..

[30] Spiliopoulou P., Spear S., Mirza H., Garner I., McGarry L., Freile F.G., Cheng Z., Ennis D.P., Iyer N.R., McNamara S. (2022). Dual G9A/EZH2 inhibition stimulates anti-tumour immune response in ovarian high grade serous carcinoma. Mol. Cancer Ther..

