# Supplementary material for: Interplay between ceRNA and Epigenetic Control of microRNA: Modelling Approaches with Application to the Role of Estrogen in Ovarian Cancer
Source: Int J Mol Sci. 2022 Feb 18;23(4):2277. doi: 10.3390/ijms23042277 (PMC8876507; doi:10.3390/ijms23042277)
Supplement: Supplementary file 1 [file ijms-23-02277-s001.zip › Supplementary Text/Supplementary Text S2.pdf]

Supplementary Text S2

In this supplement, we gives the notations of genes, complexes, rate functions, and parameter values. The descriptions and the references for the interactions in this figure are illustrated in Table S1.

Table of notations

Gene/Complex	Concentration	Transcription rate or Translation rate or Association rate	Degradation rate	Dissociation rate
miR193a	R_{mi}	k_{mi}	δ_{mi}	
E2F6 mRNA	R_1	k_1	δ_1	
cKit mRNA	R_2	k_2	δ_2	
PBX1 mRNA	R_3	k_3	δ_3	
E2F6 Protein	P_1	k_{P_1}	δ_{P_1}	
miR193a – E2F6 mRNA	C_1	$k^+_{C_1}$	δ_{C_1}	$k^-_{C_1}$
miR193a – cKit mRNA	C_2	$k^+_{C_2}$	δ_{C_2}	$k^-_{C_2}$
miR193a – PBX1 mRNA	C_3	$k^+_{C_3}$	δ_{C_3}	$k^-_{C_3}$

Description of rate functions

We assume that the transcription rate of miR193a is inhibited by the E2F6 protein in the following form

$$r_{R_{mi}} = \frac{k_{R_{mi}}}{1 + KP_1},$$

for some positive constant K. The other rate functions are given in the following ($j = 1, 2, 3$):

$$r_{R_{mi}} = \frac{k_{mi}}{1 + KP_1}, \quad r^*_{R_{mi}} = \delta_{mi} R_{mi},$$
$$r_j = k_j, \quad r^*_j = \delta_j R_j,$$
$$r_{P_1} = k_{P_1} R_1, \quad r^*_{P_1} = \delta_{P_1} P_1,$$
$$r^+_{C_j} = k^+_{C_j} R_j R_{mi}, \quad r^-_{C_j} = k^-_{C_j} C_j, \quad r^*_{C_j} = \delta_{C_j} C_j.$$

Table S1: Description of the biological function of each process in Fig 1

Interaction in Fig 1	Description of Function	References
1 (E2F6 DNA→RNA)	"E2F6 DNA transcribes to E2F6 mRNA."	
2 (c-KIT DNA→RNA)	"c-KIT DNA transcribes to c-KIT mRNA."	
3 (PBX1 DNA→RNA)	"PBX1 DNA transcribes to c-KIT mRNA."	
4 (miR193a DNA→RNA)	"miR193a DNA transcribes to miR193a RNA."	
5 (miR193a--|E2F6)	"miR193a inhibits translation of E2F6."	[1]
6 (miR193a--|c-KIT)	"miR193a inhibits translation of c-KIT."	[2, 3]
7 (miR193a--|PBX1)	"miR193a inhibits translation of PBX1."	Proposed
8 (E2F6--|miR193a)	"Inhibiting endogenous miR193a by E2F6 over-expression, which acted like a sponge in absorbing the miR193a."	[4]
9 (c-KIT --|miR193a)	"Inhibiting endogenous miR193a by -KIT over-expression, which acted like a sponge in absorbing the miR193a."	[4]
10 (PBX1 --|miR193a)	"Inhibiting endogenous miR193a by PBX1 over-expression, which acted like a sponge in absorbing the miR193a."	Proposed
11 (E2F6 RNA→protein)	"E2F6 RNA translates to E2F6 protein."	
12 (E2F6--|miR193a)	"E2F6 protein may lead to epigenetic silencing of miR-193a."	[4-7]

Parameter values

The following parameter values are taken from Osella, Bosia, Cora, and Caselle (PLoS computational biology, 2011).
Synthesis-related rates:

miR193a transcription rate: $k_{mi} = 0.3$ molecule/sec.

c-Kit mRNA transcription rate: $k_2 = 0.1$ molecule/sec.

PBX1 mRNA transcription rate: $k_3 = 0.1$ molecule/sec.

E2F6 protein translation rate: $k_{P_1} = 0.03$ molecule/sec.

- ∘ E2F6 mRNA transcription rate: k_1.
- ∘ Inhibition strength of miR193a expression: K.

} control/bifurcation parameters.

Degradation rates:

miR193a degradation rate: $\delta_{mi} = 0.0001$.

E2F6 mRNA degradation rate: $\delta_1 = 0.0003$.

c-Kit mRNA degradation rate: $\delta_2 = 0.0003$.

PBX1 mRNA degradation rate: $\delta_3 = 0.0003$.

miR193a-E2F6 mRNA complex degradation rate: $\delta_{C_1} = 0.003$.

miR193a-c-Kit mRNA complex degradation rate: $\delta_{C_2} = 0.003$.

miR193a-PBX1 mRNA complex degradation rate: $\delta_{C_3} = 0.003$.

E2F6 protein degradation rate: $\delta_{P_1} = 0.0001$.

Complexes - binding and unbinding rates:

miR193a & E2F6 mRNA association rate: $k^+_{C_1} = 0.003$.

miR193a & c-Kit mRNA association rate: $k^+_{C_2} = 0.003$.

miR193a & PBX1 mRNA association rate: $k^+_{C_3} = 0.003$.

miR193a-E2F6 mRNA complex dissociation rate: $k^-_{C_1} = 0.001$.

miR193a-c-Kit mRNA complex dissociation rate: $k^-_{C_2} = 0.001$.

miR193a-E2F6 mRNA complex dissociation rate: $k^-_{C_3} = 0.001$.

References

1. Kozaki, K.; Imoto, I.; Mogi, S.; Omura, K.; Inazawa, J., Exploration of tumor-suppressive microRNAs silenced by DNA hypermethylation in oral cancer. *Cancer Res* **2008,** 68, (7), 2094-105.
2. Gao, X. N.; Lin, J.; Li, Y. H.; Gao, L.; Wang, X. R.; Wang, W.; Kang, H. Y.; Yan, G. T.; Wang, L. L.; Yu, L., MicroRNA-193a represses c-kit expression and functions as a methylation-silenced tumor suppressor in acute myeloid leukemia. *Oncogene* **2011,** 30, (31), 3416-28.
3. Li, Y.; Gao, L.; Luo, X.; Wang, L.; Gao, X.; Wang, W.; Sun, J.; Dou, L.; Li, J.; Xu, C.; Wang, L.; Zhou, M.; Jiang, M.; Zhou, J.; Caligiuri, M. A.; Nervi, C.; Bloomfield, C. D.; Marcucci, G.; Yu, L., Epigenetic silencing of microRNA-193a contributes to leukemogenesis in t(8;21) acute myeloid leukemia by activating the PTEN/PI3K signal pathway. *Blood* **2013,** 121, (3), 499-509.
4. Cheng, F. H. C.; Lin, H. Y.; Hwang, T. W.; Chen, Y. C.; Huang, R. L.; Chang, C. B.; Yang, W.; Lin, R. I.; Lin, C. W.; Chen, G. C. W.; Mai, S. Y.; Lin, J. M. J.; Chuang, Y. M.; Chou, J. L.; Kuo, L. W.; Li, C.; Cheng, A. S. L.; Lai, H. C.; Wu, S. F.; Tsai, J. C.; Chan, M. W. Y., E2F6 functions as a competing endogenous RNA, and transcriptional repressor, to promote ovarian cancer stemness. *Cancer Sci* **2019,** 110, (3), 1085-1095.
5. Trimarchi, J. M.; Fairchild, B.; Wen, J.; Lees, J. A., The E2F6 transcription factor is a component of the mammalian Bmi1-containing polycomb complex. *Proc Natl Acad Sci U S A* **2001,** 98, (4), 1519-24.
6. Attwooll, C.; Oddi, S.; Cartwright, P.; Prosperini, E.; Agger, K.; Steensgaard, P.; Wagener, C.; Sardet, C.; Moroni, M. C.; Helin, K., A novel repressive E2F6 complex containing the polycomb group protein, EPC1, that interacts with EZH2 in a proliferation-specific manner. *J Biol Chem* **2005,** 280, (2), 1199-208.
7. Velasco, G.; Hube, F.; Rollin, J.; Neuillet, D.; Philippe, C.; Bouzinba-Segard, H.; Galvani, A.; Viegas-Pequignot, E.; Francastel, C., Dnmt3b recruitment through E2F6 transcriptional repressor mediates germ-line gene silencing in murine somatic tissues. *Proc Natl Acad Sci U S A* **2010,** 107, (20), 9281-6.
8. Osella, M.; Bosia, C.; Cora, D.; Caselle, M., The role of incoherent microRNA-mediated feedforward loops in noise buffering. *Proc Natl Acad Sci U S APLoS computational biology* **2011,** 7, (3), e1001101.
