# Supplementary material for: Interplay between ceRNA and Epigenetic Control of microRNA: Modelling Approaches with Application to the Role of Estrogen in Ovarian Cancer
Source: Int J Mol Sci. 2022 Feb 18;23(4):2277. doi: 10.3390/ijms23042277 (PMC8876507; doi:10.3390/ijms23042277)
Supplement: Supplementary file 1 [file ijms-23-02277-s001.zip › Supplementary Text/Supplementary Text S1.pdf]

Supplementary Text S1

In this supplement, we will mathematically show that for the case that the miRNA R_{mi} has multiple targeted mRNAs, say R_1, R_2, $\ldots$, R_n, the transcriptional inhibition of R_{mi} by R_1-translated protein P_1 is necessary for the rapid increase in the other mRNA levels. The strategy is to mathematically show that the transcriptional inhibition of R_{mi} by P_1 is necessary for the occurrence of (saddle-node) bifurcations which would lead to the significant increase of targeted mRNA levels.

1 The setting

For convenience, we use the following notation. Let $\mathbb{R}^{p\times q}$ be the space of all real $p \times q$ matrices, and put

$$\mathbf{0}_p = [0]_{p\times 1},\ \mathbf{1}_p = [1]_{p\times 1},\ \mathbf{e}_p = \begin{bmatrix} 1 \\ 0 \\ \vdots \\ 0 \end{bmatrix} \in \mathbb{R}^{p\times 1},\ \mathbf{O}_p = [0]_{p\times p} \in \mathbb{R}^{p\times p},\ \mathbf{O}_{p\times q} = [0]_{p\times q} \in \mathbb{R}^{p\times q},$$

and let I_p be the $p \times p$ identity matrix, for all positive integer p, q.

Consider the following system of ordinary differential equations which is a generalization of system (1) in the main text:

$$\frac{d}{dt}\mathbf{x}(t) \quad = \quad \boldsymbol{\nu} \cdot \boldsymbol{r}\left(\mathbf{x}(t)\right) \quad \equiv \quad \mathbf{f}\left(\mathbf{x}(t)\right),$$

where $\mathbf{x} = [x_1, \cdots, x_M]^\top$, $Y = [r_1, \cdots, r_{M+k}]^\top$, $\mathbf{f} = [f_1, \cdots, f_M]^\top$, and

$$\boldsymbol{\nu} = \left[\begin{array}{cccc|ccc} \nu_{11} & \nu_{12} & \cdots & \nu_{1\,M} & \nu_{1\,M+1} & \cdots & \nu_{1\,M+k} \\ \nu_{21} & \nu_{22} & \cdots & \nu_{2\,M} & \nu_{2\,M+1} & \cdots & \nu_{2\,M+k} \\ \vdots & \vdots & \vdots & \vdots & \vdots & \vdots & \vdots \\ \nu_{M\,1} & \nu_{M\,2} & \cdots & \nu_{M\,M} & \nu_{M\,M+1} & \cdots & \nu_{M\,M+k} \end{array}\right]_{M\times(M+k)} \equiv [\,L\,|\,B\,],$$

where $L \in \mathbb{R}^{M\times M}$, $B \in \mathbb{R}^{M\times k}$, and M and $M+k$ are the total numbers of genes and rate functions involved in the network system, respectively. Here $\boldsymbol{\nu}$ and $\boldsymbol{r}$ are the stoichiometric matrix and the reaction rate functions/kinetics of the network system, respectively. Their exact forms will be specified later. We also write $\boldsymbol{r}$ as

$$\boldsymbol{r} = \begin{bmatrix} \bar{\mathbf{r}} \\ \hat{\mathbf{r}} \end{bmatrix},$$

where $\bar{\mathbf{r}} = [r_1, \ldots, r_M]^\top$ and $\hat{\mathbf{r}} = [r_{M+1}, \ldots, r_{M+k}]^\top$.

Next, we will introduce a matrix A and show that A has zero eigenvalues whenever the Jacobian matrix $D_{\mathbf{x}}\mathbf{f}$ does. To proceed, we gives the null space of the stoichiometric matrix $\boldsymbol{\nu}$ in the following lemma.

Lemma 1. *Assume that L is invertible. Let $N(\boldsymbol{\nu})$ be the null space of $\boldsymbol{\nu}$. Then we have*

$$N(\boldsymbol{\nu}) = \textit{The column space of} \left[\begin{array}{c} -L^{-1}\cdot B \\ \hline I_{k\times k} \end{array}\right]_{(M+k)\times k}.$$

Proof. Let $\boldsymbol{r} = \begin{bmatrix} \bar{\mathbf{r}} \\ \hat{\mathbf{r}} \end{bmatrix} \in N(\boldsymbol{\nu}) \subseteq \mathbb{R}^{(M+k)\times 1}$, where $\bar{\mathbf{r}} \in \mathbb{R}^{M\times 1}$ and $\hat{\mathbf{r}} \in \mathbb{R}^{k\times 1}$. Then one has $L\bar{\mathbf{r}} + B\hat{\mathbf{r}} = \mathbf{0}_{M+k}$, which can be written as $\bar{\mathbf{r}} = -L^{-1}B\hat{\mathbf{r}}$. Or equivalently,

$$\boldsymbol{r} = Q\hat{\mathbf{r}} := \left[\begin{array}{c} -L^{-1}\cdot B \\ \hline I_{k\times k} \end{array}\right]\hat{\mathbf{r}},$$

which implies that $N(\boldsymbol{\nu})$ is contained in the column space of Q.

Note that the above argument can be reversed to obtain that the column space of Q is a subset of $N(\boldsymbol{\nu})$. Thus, the proof is completed. ∎

Now, introduce the matrix

$$A = \begin{bmatrix} D_{\mathbf{x}}\,\bar{\mathbf{r}} & -L^{-1}B \\ D_{\mathbf{x}}\,\hat{\mathbf{r}} & I_{k\times k} \end{bmatrix} \in \mathbb{R}^{(M+k)\times(M+k)}.$$

The A matrix was introduced by Mochizuki and Fiedler [1] for the sensitivities of the chemicals. Here, for ease of the analysis, we use the basis given in Lemma 1 for the null space of the stoichiometric matrix ν. The following lemma gives the link between the A matrix and the Jacobian matrix $D_{\mathbf{x}}\mathbf{f}$.

Proposition 2. *Assume that L is invertible. Then we have*

$$\det A = \det\left(D_{\mathbf{x}}\,\bar{\mathbf{r}} + L^{-1}BD_{\mathbf{x}}\,\hat{\mathbf{r}}\right) = (\det L)^{-1}\cdot\det D_{\mathbf{x}}\mathbf{f}(\mathbf{x}). \tag{1}$$

In particular, $\det A = 0$ *if and only if* $\det D_{\mathbf{x}}\,\mathbf{f}(\mathbf{x}) = 0$.

Proof. First, from the identity

$$\mathbf{f}(\mathbf{x}) = \boldsymbol{\nu}\,\boldsymbol{r}(\mathbf{x}) = L\,\bar{\mathbf{r}}(\mathbf{x}) + B\,\hat{\mathbf{r}}(\mathbf{x}),$$

it follows that

$$D_{\mathbf{x}}\mathbf{f}(\mathbf{x}) = L\,D_{\mathbf{x}}\bar{\mathbf{r}}(\mathbf{x}) + B\,D_{\mathbf{x}}\hat{\mathbf{r}}(\mathbf{x}) = L\big(D_{\mathbf{x}}\bar{\mathbf{r}}(\mathbf{x}) + L^{-1}B\,D_{\mathbf{x}}\hat{\mathbf{r}}(\mathbf{x})\big). \tag{2}$$

Next, observe that

$$\begin{bmatrix} I_{M\times M} & L^{-1}B \\ \mathbf{O}_{k\times M} & I_{k\times k} \end{bmatrix}\cdot A = \begin{bmatrix} D_{\mathbf{x}}\,\bar{\mathbf{r}} + L^{-1}BD_{\mathbf{x}}\,\hat{\mathbf{r}} & \mathbf{0}_{M\times k} \\ D_{\mathbf{x}}\,\hat{\mathbf{r}} & I_{k\times k} \end{bmatrix},$$

from which the identity

$$\det A = \det\left(D_{\mathbf{x}}\,\bar{\mathbf{r}} + L^{-1}BD_{\mathbf{x}}\,\hat{\mathbf{r}}\right) = (\det L)^{-1}\cdot\det D_{\mathbf{x}}\,\mathbf{f}(\mathbf{x})$$

follows. Here, (2) is used in the second equality. This completes the proof. ∎

2 The proof

Reaction Kinetics: First, the reaction scheme of the network system can be depicted as follows:

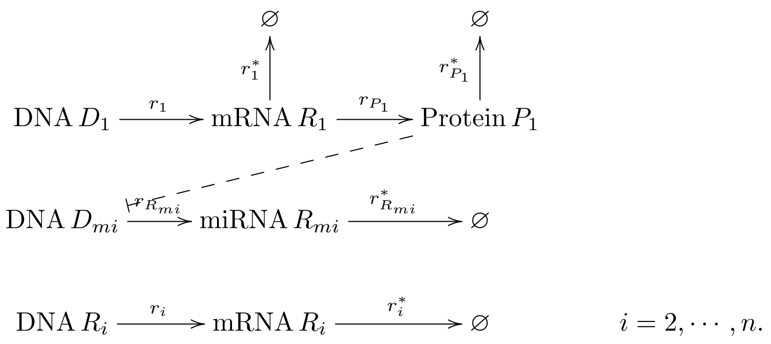

$$R_{mi} + R_i \underset{r^-_{C_i}}{\overset{r^+_{C_i}}{\rightleftharpoons}} C_i \xrightarrow{r^*_{C_i}} \varnothing \qquad i = 1, 2, \cdots, n.$$

We remark that the transcriptional inhibition of R_{mi} by protein P_1 corresponds to the inequality

$$\partial_{P_1} r_{R_{mi}} < 0.$$

Table of notations:

Complex	Concentration	Transcription rate or Translation rate or Association rate	Degradation rate	Dissociation rate
miRNA R_{mi}	R_{mi}	$r_{R_{mi}}$	$r^*_{R_{mi}}$	
mRNA R_i	R_i	r_i	r^*_i	
Protein P_1	P	r_{P_1}	$r^*_{P_1}$	
miRNA R_{mi} + mRNA R_i	C_i	$r^+_{C_i}$	$r^*_{C_i}$	$r^-_{C_i}$

In the reminder of this appendix, we will prove that if the targeted protein P_1 does not modulate the transcription rate of the miRNA R_{mi}, then the Jacobian matrix $D_{\mathbf{x}}\,\mathbf{f}$ of the network system is invertible. This in turn implies that there will be no occurrences of bifurcations, and hence that the levels of mRNA R_i's will not be significantly changed.

Now we list the reaction steps corresponding to the reaction scheme as follows:

Reactors		Products	P_1	R_{mi}	R_1	$\cdots$	R_n	C_1	$\cdots$	C_n	Reaction Rate
R_1	$\to$	P_1	1	0	0	$\cdots$	0	0	$\cdots$	0	r_{P_1}
$\varnothing$	$\to$	R_{mi}	0	1	0	$\cdots$	0	0	$\cdots$	0	$r_{R_{mi}}$
$\varnothing$	$\to$	R_1	0	0	1	$\cdots$	0	0	$\cdots$	0	r_1
$\vdots$	$\vdots$	$\vdots$	$\vdots$	$\vdots$	$\vdots$	$\vdots$	$\vdots$	$\vdots$	$\vdots$	$\vdots$	$\vdots$
$\varnothing$	$\to$	R_n	0	0	0	$\cdots$	1	0	$\cdots$	0	r_n
$R_{mi} + R_1$	$\to$	C_1	0	-1	-1	$\cdots$	0	1	$\cdots$	0	$r^+_{C_1}$
$\vdots$	$\vdots$	$\vdots$	$\vdots$	$\vdots$	$\vdots$	$\vdots$	$\vdots$	$\vdots$	$\vdots$	$\vdots$	$\vdots$
$R_{mi} + R_n$	$\to$	C_n	0	-1	0	$\cdots$	-1	0	$\cdots$	1	$r^+_{C_n}$
P_1	$\to$	$\varnothing$	-1	0	0	$\cdots$	0	0	$\cdots$	0	$r^*_{P_1}$
R_{mi}	$\to$	$\varnothing$	0	-1	0	$\cdots$	0	0	$\cdots$	0	$r^*_{R_{mi}}$
R_1	$\to$	$\varnothing$	0	0	-1	$\cdots$	0	0	$\cdots$	0	r^*_1
$\vdots$	$\vdots$	$\vdots$	$\vdots$	$\vdots$	$\vdots$	$\vdots$	$\vdots$	$\vdots$	$\vdots$	$\vdots$	$\vdots$
R_n	$\to$	$\varnothing$	0	0	0	$\cdots$	-1	0	$\cdots$	0	r^*_n
C_1	$\to$	$\varnothing$	0	0	0	$\cdots$	0	-1	$\cdots$	0	$r^*_{C_1}$
$\vdots$	$\vdots$	$\vdots$	$\vdots$	$\vdots$	$\vdots$	$\vdots$	$\vdots$	$\vdots$	$\vdots$	$\vdots$	$\vdots$
C_n	$\to$	$\varnothing$	0	0	0	$\cdots$	0	0	$\cdots$	-1	$r^*_{C_n}$
C_1	$\to$	$R_{mi} + R_1$	0	1	1	$\cdots$	0	-1	$\cdots$	0	$r^-_{C_1}$
$\vdots$	$\vdots$	$\vdots$	$\vdots$	$\vdots$	$\vdots$	$\vdots$	$\vdots$	$\vdots$	$\vdots$	$\vdots$	$\vdots$
C_n	$\to$	$R_{mi} + R_n$	0	1	0	$\cdots$	1	0	$\cdots$	-1	$r^-_{C_n}$

Then the stoichiometric matrix is given by $\boldsymbol{\nu} = [L \,|\, B] \in \mathbb{R}^{(2n+2)\times(5n+4)}$, with $M = 2n+2$ and $k = 3n+2$, where the matrices L and B are, respectively, defined by

$$L = \begin{bmatrix} 1 & 0 & \mathbf{0}_n^\top & \mathbf{0}_n^\top \\ 0 & 1 & \mathbf{0}_n^\top & -\mathbf{1}_n^\top \\ \mathbf{0}_n & \mathbf{0}_n & I_n & -I_n \\ \mathbf{0}_n & \mathbf{0}_n & \mathbf{O}_n & I_n \end{bmatrix} \;\&\; B = \begin{bmatrix} -1 & 0 & \mathbf{0}_n^\top & \mathbf{0}_n^\top & \mathbf{0}_n^\top \\ 0 & -1 & \mathbf{0}_n^\top & \mathbf{0}_n^\top & \mathbf{1}_n^\top \\ \mathbf{0}_n & \mathbf{0}_n & -I_n & \mathbf{O}_n & I_n \\ \mathbf{0}_n & \mathbf{0}_n & \mathbf{O}_n & -I_n & -I_n \end{bmatrix}.$$

Thus L is invertible. Moreover, we have

$$L^{-1} = \begin{bmatrix} 1 & 0 & \mathbf{0}_n^\top & \mathbf{0}_n^\top \\ 0 & 1 & \mathbf{0}_n^\top & \mathbf{1}_n^\top \\ \mathbf{0}_n & \mathbf{0}_n & I_n & I_n \\ \mathbf{0}_n & \mathbf{0}_n & \mathbf{O}_n & I_n \end{bmatrix} \;\&\; L^{-1}B = \begin{bmatrix} -1 & 0 & \mathbf{0}_n^\top & \mathbf{0}_n^\top & \mathbf{0}_n^\top \\ 0 & -1 & \mathbf{0}_n^\top & -\mathbf{1}_n^\top & \mathbf{0}_n^\top \\ \mathbf{0}_n & \mathbf{0}_n & -I_n & -I_n & \mathbf{O}_n \\ \mathbf{0}_n & \mathbf{0}_n & \mathbf{O}_n & -I_n & -I_n \end{bmatrix}.$$

Put

$$\mathbf{r} = \begin{bmatrix} r_1 \\ \vdots \\ r_n \end{bmatrix}, \mathbf{r}^* = \begin{bmatrix} r^* \\ \vdots \\ r^* \end{bmatrix}, \mathbf{r}_{\mathbf{C}}^+ = \begin{bmatrix} r_{C_1}^+ \\ \vdots \\ r_{C_n}^+ \end{bmatrix}, \mathbf{r}_{\mathbf{C}}^- = \begin{bmatrix} r_{C_1}^- \\ \vdots \\ r_{C_n}^- \end{bmatrix}, \mathbf{r}_{\mathbf{C}}^* = \begin{bmatrix} r_{C_1}^* \\ \vdots \\ r_{C_n}^* \end{bmatrix},$$

and

$$\mathbf{x} = \begin{bmatrix} P_1 \\ R_{mi} \\ R_1 \\ \vdots \\ R_n \\ C_1 \\ \vdots \\ C_n \end{bmatrix}, \bar{\mathbf{r}} = \begin{bmatrix} r_{P_1} \\ r_{R_{mi}} \\ \mathbf{r} \\ \mathbf{r}_{\mathbf{C}}^+ \end{bmatrix} \in \mathbb{R}^M, \hat{\mathbf{r}} = \begin{bmatrix} r_{P_1}^* \\ r_{R_{mi}}^* \\ \mathbf{r}^* \\ \mathbf{r}_{\mathbf{C}}^* \\ \mathbf{r}_{\mathbf{C}}^- \end{bmatrix} \in \mathbb{R}^k.$$

We will compute $\det\left(D_{\mathbf{x}}\,\bar{\mathbf{r}} + L^{-1}BD_{\mathbf{x}}\,\hat{\mathbf{r}}\right)$. The computation is divided into three steps.

Computation of $D_{\mathbf{x}}\bar{\mathbf{r}}$:

Indeed, we have

$$D_{\mathbf{x}}\bar{\mathbf{r}} = D_{\mathbf{x}} \begin{bmatrix} r_{P_1} \\ r_{R_{mi}} \\ \mathbf{r} \\ \mathbf{r}_{\mathbf{C}}^+ \end{bmatrix} = \begin{bmatrix} 0 & 0 & (\partial_{R_1} r_{P_1})\,\mathbf{e}_n^\top & \mathbf{0}_n^\top \\ \partial_{P_1} r_{R_{mi}} & 0 & \mathbf{0}_n^\top & \mathbf{0}_n^\top \\ \mathbf{0}_n & \mathbf{0}_n & \mathbf{O}_n & \mathbf{O}_n \\ \mathbf{0}_n & \partial_{R_{mi}}\mathbf{r}_{\mathbf{C}}^+ & \Delta & \mathbf{O}_n \end{bmatrix} \in \mathbb{R}^{(2n+2)\times(2n+2)}.$$

Here,

$$\partial_{R_{mi}}\mathbf{r}_{\mathbf{C}}^+ = \begin{bmatrix} \partial_{R_{mi}} r_{C_1}^+ \\ \vdots \\ \partial_{R_{mi}} r_{C_n}^+ \end{bmatrix} \in \mathbb{R}^n \text{ and } \Delta = \begin{bmatrix} \partial_{R_1} r_{C_1}^+ & 0 & \cdots & 0 \\ 0 & \partial_{R_2} r_{C_2}^+ & \cdots & 0 \\ \vdots & \vdots & \ddots & \vdots \\ 0 & 0 & \cdots & \partial_{R_n} r_{C_n}^+ \end{bmatrix} \in \mathbb{R}^{n\times n}.$$

Computation of $D_{\mathbf{x}}\bar{\mathbf{r}} + L^{-1}B\left(D_{\mathbf{x}}\hat{\mathbf{r}}\right)$:

On the other hand, one has

$$D_{\mathbf{x}}\hat{\mathbf{r}} = \begin{bmatrix} J_1 & \mathbf{O}_{2\times n} & \mathbf{O}_{2\times n} \\ \mathbf{O}_{n\times 2} & J_2 & \mathbf{O}_n \\ \mathbf{O}_{n\times 2} & \mathbf{O}_n & J_3 \\ \mathbf{O}_{n\times 2} & \mathbf{O}_n & J_4 \end{bmatrix}$$

where

$$J_1 = \begin{bmatrix} \partial_{P_1} r_{P_1}^* & 0 \\ 0 & \partial_{R_{mi}} r_{R_{mi}}^* \end{bmatrix}, J_2 = \begin{bmatrix} \partial_{R_1} r_1^* & 0 & 0 & \cdots & 0 \\ 0 & \partial_{R_2} r_2^* & 0 & \cdots & 0 \\ \vdots & \vdots & \ddots & \vdots & \vdots \\ 0 & 0 & 0 & \cdots & \partial_{R_n} r_n^* \end{bmatrix} \in \mathbb{R}^{n\times n},$$

$$J_3 = \begin{bmatrix} \partial_{C_1} r^*_{C_1} & 0 & 0 & \cdots & 0 \\ 0 & \partial_{C_2} r^*_{C_2} & 0 & \cdots & 0 \\ \vdots & \vdots & \ddots & \vdots & \vdots \\ 0 & 0 & 0 & \cdots & \partial_{C_n} r^*_{C_n} \end{bmatrix}, \text{ and } J_4 = \begin{bmatrix} \partial_{C_1} r^-_{C_1} & 0 & 0 & \cdots & 0 \\ 0 & \partial_{C_2} r^-_{C_2} & 0 & \cdots & 0 \\ \vdots & \vdots & \ddots & \vdots & \vdots \\ 0 & 0 & 0 & \cdots & \partial_{C_n} r^-_{C_n} \end{bmatrix} \in \mathbb{R}^{n\times n}.$$

Accordingly,

$$\begin{aligned} L^{-1}B\left(D_{\mathbf{x}}\hat{\mathbf{r}}\right) &= \begin{bmatrix} -I_2 & \mathbf{O}_{2\times n} & -V & \mathbf{O}_{2\times n} \\ \mathbf{O}_{n\times 2} & -I_n & -I_n & \mathbf{O}_n \\ \mathbf{O}_{n\times 2} & \mathbf{O}_n & -I_n & -I_n \end{bmatrix} \begin{bmatrix} J_1 & \mathbf{O}_{2\times n} & \mathbf{O}_{2\times n} \\ \mathbf{O}_{n\times 2} & J_2 & \mathbf{O}_n \\ \mathbf{O}_{n\times 2} & \mathbf{O}_n & J_3 \\ \mathbf{O}_{n\times 2} & \mathbf{O}_n & J_4 \end{bmatrix} \\ &= -\begin{bmatrix} J_1 & \mathbf{O}_{2\times n} & VJ_3 \\ \mathbf{O}_{n\times 2} & J_2 & J_3 \\ \mathbf{O}_{n\times 2} & \mathbf{O}_n & J_3+J_4 \end{bmatrix}, \end{aligned}$$

where $V = \begin{bmatrix} \mathbf{0}_n^\top \\ \mathbf{1}_n^\top \end{bmatrix} \in \mathbb{R}^{2\times n}$. Taken together, it follows that

$$\begin{aligned} D_{\mathbf{x}}\bar{\mathbf{r}} + L^{-1}B\left(D_{\mathbf{x}}\hat{\mathbf{r}}\right) &= \begin{bmatrix} 0 & 0 & \left(\partial_{R_1} r_{P_1}\right)\mathbf{e}_n^\top & \mathbf{0}_n^\top \\ \partial_{P_1} r_{R_{mi}} & 0 & \mathbf{0}_n^\top & \mathbf{0}_n^\top \\ \mathbf{0}_n & \mathbf{0}_n & \mathbf{O}_n & \mathbf{O}_n \\ \mathbf{0}_n & \partial_{R_{mi}}\mathbf{r}^+_{\mathbf{C}} & \Delta & \mathbf{O}_n \end{bmatrix} - \begin{bmatrix} J_1 & \mathbf{O}_{2\times n} & VJ_3 \\ \mathbf{O}_{n\times 2} & J_2 & J_3 \\ \mathbf{O}_{n\times 2} & \mathbf{O}_n & J_3+J_4 \end{bmatrix} \\ &= \begin{bmatrix} 0 & 0 & \left(\partial_{R_1} r_{P_1}\right)\mathbf{e}_n^\top & \mathbf{0}_n^\top \\ \partial_{P_1} r_{R_{mi}} & 0 & \mathbf{0}_n^\top & \mathbf{0}_n^\top \\ \mathbf{0}_n & \mathbf{0}_n & \mathbf{O}_n & \mathbf{O}_n \\ \mathbf{0}_n & \partial_{R_{mi}}\mathbf{r}^+_{\mathbf{C}} & \Delta & \mathbf{O}_n \end{bmatrix} - \begin{bmatrix} r^*_{P_1} & 0 & \mathbf{0}_n^\top & \mathbf{0}_n^\top \\ 0 & r^*_{R_{mi}} & \mathbf{0}_n^\top & \mathbf{1}_n^\top J_3 \\ \mathbf{0}_n & \mathbf{0}_n & J_2 & J_3 \\ \mathbf{0}_n & \mathbf{0}_n & \mathbf{O}_n & J_3+J_4 \end{bmatrix} \\ &= -\begin{bmatrix} r^*_{P_1} & 0 & -\left(\partial_{R_1} r_{P_1}\right)\mathbf{e}_n^\top & \mathbf{0}_n^\top \\ -\partial_{P_1} r_{R_{mi}} & r^*_{R_{mi}} & \mathbf{0}_n^\top & \mathbf{1}_n^\top J_3 \\ \mathbf{0}_n & \mathbf{0}_n & J_2 & J_3 \\ \mathbf{0}_n & -\partial_{R_{mi}}\mathbf{r}^+_{\mathbf{C}} & -\Delta & J_3+J_4 \end{bmatrix}. \end{aligned}$$

Computation of $\det\left(D_{\mathbf{x}}\bar{\mathbf{r}} + L^{-1}B\left(D_{\mathbf{x}}\hat{\mathbf{r}}\right)\right)$:

Using the above computations, we arrive at the following crucial identity:

$$\begin{aligned} & \det\left(D_{\mathbf{x}}\bar{\mathbf{r}} + L^{-1}B\left(D_{\mathbf{x}}\hat{\mathbf{r}}\right)\right) \\ = \quad & r^*_{P_1}\cdot\det\begin{bmatrix} r^*_{R_{mi}} & \mathbf{0}_n^\top & \mathbf{1}_n^\top J_3 \\ \mathbf{0}_n & J_2 & J_3 \\ -\partial_{R_{mi}}\mathbf{r}^+_{\mathbf{C}} & -\Delta & J_3+J_4 \end{bmatrix} + \partial_{P_1} r_{R_{mi}}\cdot\det\begin{bmatrix} 0 & -\left(\partial_{R_1} r_{P_1}\right)\mathbf{e}_n^\top & \mathbf{0}_n^\top \\ \mathbf{0}_n & J_2 & J_3 \\ -\partial_{R_{mi}}\mathbf{r}^+_{\mathbf{C}} & -\Delta & J_3+J_4 \end{bmatrix} \quad (3) \\ := \quad & r^*_{P_1}\Gamma_1 + \partial_{P_1} r_{R_{mi}}\Gamma_2. \end{aligned}$$

Note that Γ_1 can be written as

$$\Gamma_1 = \begin{bmatrix} J_2^* & C \\ -H & J^* \end{bmatrix},$$

where $J_2^* = \begin{bmatrix} r^*_{R_{mi}} & \mathbf{0}_n^\top \\ \mathbf{0}_n & J_2 \end{bmatrix} \in \mathbb{R}^{(n+1)\times(n+1)}$, $J^* = J_3 + J_4 \in \mathbb{R}^{n\times n}$, $H = \begin{bmatrix} \partial_{R_{mi}} \mathbf{r}^+_{\mathbf{C}} & \Delta \end{bmatrix} \in \mathbb{R}^{n\times(n+1)}$ and $C = \begin{bmatrix} \mathbf{1}_n^\top J_3 \\ J_3 \end{bmatrix} \in \mathbb{R}^{(n+1)\times n}$. In the following two lemmas, we will show that $\det(\Gamma_1) > 0$ and $\det(\Gamma_2) > 0$. This, together with (1), (3) and the fact that $r^*_{P_1} > 0$, yields the main assertion that the transcriptional inhibition of R_{mi} by protein P_1 (i.e., $\partial_{P_1} r_{R_{mi}} < 0$) is necessary for the occurrence of (saddle-node) bifurcation, and so the significant increase of target mRNAs.

The remainder of this supporting material is to verify that $\det(\Gamma_1) > 0$ and $\det(\Gamma_2) > 0$.

Lemma 3. *We have* $\det \Gamma_1 > 0$.

Proof. Since

$$\begin{bmatrix} I_{n+1} & \mathbf{O}_{(n+1)\times n} \\ H & I_n \end{bmatrix} \begin{bmatrix} (J_2^*)^{-1} & \mathbf{O}_{(n+1)\times n} \\ \mathbf{O}_{n\times(n+1)} & I_n \end{bmatrix} \Gamma_1 = \begin{bmatrix} I_{n+1} & (J_2^*)^{-1}C \\ \mathbf{O}_{(n+1)\times n} & H(J_2^*)^{-1}C + J^* \end{bmatrix},$$

one has

$$\det \Gamma_1 = (\det(J_2^*)^{-1})^{-1} \cdot \det(H(J_2^*)^{-1}C + J^*).$$

Let $H^* = H(J_2^*)^{-1} = [\mathbf{y},\ \Delta^*]$, where $\mathbf{y} = \frac{1}{r^*_{R_{mi}}} \partial_{R_{mi}} \mathbf{r}^+_{\mathbf{C}} \in \mathbb{R}^{n\times 1}$ and

$$\Delta^* = \begin{bmatrix} \frac{\partial_{R_1} r^+_{C_1}}{\partial_{R_1} r^*_1} & 0 & \cdots & 0 \\ 0 & \frac{\partial_{R_2} r^+_{C_2}}{\partial_{R_2} r^*_2} & \cdots & 0 \\ \vdots & \vdots & \ddots & \vdots \\ 0 & 0 & \cdots & \frac{\partial_{R_n} r^+_{C_n}}{\partial_{R_n} r^*_n} \end{bmatrix} \in \mathbb{R}^{n\times n}.$$

Put $\mathbf{z} = \begin{bmatrix} \partial_{C_1} r^*_{C_1} \\ \vdots \\ \partial_{C_n} r^*_{C_n} \end{bmatrix}$, that is, $\mathbf{z}^\top = \mathbf{1}_n^\top J_3$. Therefore, we have

$$H(J_2^*)^{-1}C + J^* = \mathbf{y}\mathbf{z}^\top + \Delta^* \cdot J_3 + J^* = (J^* + \Delta^* \cdot J_3)\left(I + (J^* + \Delta^* \cdot J_3)^{-1} \mathbf{y}\mathbf{z}^\top\right).$$

Let $\tilde{\mathbf{y}} = (J^* + \Delta^* \cdot J_3)^{-1} \mathbf{y}$. Since $\Delta^*,\ J_3,\ J^* \in \mathbb{R}^{n\times n}$ are diagonal matrices whose diagonal entries are all positive and all entries of $\mathbf{y}$ are positive, it follows that all entries of $\tilde{\mathbf{y}}$ are also positive.

Now note that $(\mathbf{z}^\top\tilde{\mathbf{y}}, \tilde{\mathbf{y}}) \in \mathbb{R} \times (\mathbb{R}^{n\times 1} - \{\mathbf{0}_n\})$ is an eigenpair of the $n \times n$ matrix $\tilde{\mathbf{y}}\mathbf{z}^\top$. If $\mathbf{v} \in \mathbb{R}^{n\times 1} - \{\mathbf{0}_n\}$ is another eigenvector corresponding to $\mathbf{z}^\top\tilde{\mathbf{y}}$, then

$$(\mathbf{z}^\top\tilde{\mathbf{y}})\mathbf{v} = \tilde{\mathbf{y}}\mathbf{z}^\top\mathbf{v} = (\mathbf{z}^\top\mathbf{v})\tilde{\mathbf{y}}.$$

Since all entries of $\tilde{\mathbf{y}}, \mathbf{z}$ are positive, $\mathbf{z}^\top\tilde{\mathbf{y}} > 0$, which implies $\mathbf{v}$ is a constant multiple of $\tilde{\mathbf{y}}$. Consequently, the dimension of the eigenspace corresponding to $\mathbf{z}^\top\tilde{\mathbf{y}}$ is one. On the other hand, $0 \in \mathbb{R}$ is also an eigenvalue of $\tilde{\mathbf{y}}\mathbf{z}^\top$ and the corresponding eigenspace is of dimension $m - 1$. In fact, the linear space

$$E \equiv \{\mathbf{v} \in \mathbb{R}^{n\times 1} \mid \mathbf{z}^\top\mathbf{v} = 0\}$$

is the eigenspace corresponding to the eigenvalue 0. As a result, there exists an invertible matrix $T \in \mathbb{R}^{n\times n}$ such that

$$\tilde{\mathbf{y}}\mathbf{z}^\top = T \begin{bmatrix} 0 & & & \\ & \ddots & & \\ & & 0 & \\ & & & \mathbf{z}^\top\tilde{\mathbf{y}} \end{bmatrix} T^{-1},$$

from which follows

$$\det(H(J_2^*)^{-1}C + J^*) = \det(J^* + \Delta^* \cdot J_3) \cdot (1 + \mathbf{z}^\top\tilde{\mathbf{y}}) > 0.$$

The proof is thus completed. ∎

Lemma 4. *We have* $\det\Gamma_2 > 0$.

Proof. Observe that

$$\det\Gamma_2 \quad = \quad (\partial_{R_1} r_{P_1}) \det \begin{bmatrix} \tilde{J}_2 & J_3 \\ -\tilde{\Delta} & J^* \end{bmatrix},$$

where

$$\tilde{J}_2 = \begin{bmatrix} 0 & 0 & 0 & \cdots & 0 \\ 0 & \partial_{R_2} r_2^* & 0 & \cdots & 0 \\ 0 & 0 & \partial_{R_3} r_3^* & \cdots & 0 \\ \vdots & \vdots & \vdots & \ddots & \vdots \\ 0 & 0 & 0 & \cdots & \partial_{R_n} r_n^* \end{bmatrix} \quad \text{and} \quad \tilde{\Delta} = \begin{bmatrix} \partial_{R_{mi}} r_{C_1}^+ & 0 & \cdots & 0 \\ \partial_{R_{mi}} r_{C_2}^+ & \partial_{R_2} r_{C_2}^+ & \cdots & 0 \\ \vdots & \vdots & \ddots & \vdots \\ \partial_{R_{mi}} r_{C_n}^+ & 0 & \cdots & \partial_{R_n} r_{C_n}^+ \end{bmatrix}.$$

Since

$$\begin{bmatrix} I_n & -J_3 \\ \mathbf{O}_n & I_n \end{bmatrix} \begin{bmatrix} I_n & \mathbf{O}_n \\ \mathbf{O}_n & (J^*)^{-1} \end{bmatrix} \begin{bmatrix} \tilde{J}_2 & J_3 \\ -\tilde{\Delta} & J^* \end{bmatrix} = \begin{bmatrix} \tilde{J}_2 + J_3(J^*)^{-1}\tilde{\Delta} & \mathbf{O}_n \\ -(J^*)^{-1}\tilde{\Delta} & I_n \end{bmatrix}$$

and $\tilde{J}_2 + J_3(J^*)^{-1}\tilde{\Delta}$ is a lower triangular matrix whose diagonal entries are all positive, one has

$$\det\Gamma_2 = (\partial_{R_1} r_{P_1}) \left(\det(J^*)^{-1}\right)^{-1} \cdot \det\left(\tilde{J}_2 + J_3(J^*)^{-1}\tilde{\Delta}\right) > 0.$$

The proof is thus completed. ∎

References

[1] A. Mochizuki and B. Fiedler, *Sensitivity of chemical reaction networks: A structural approach. 1. Examples and the carbon metabolic network*, J. Theor. Biol. **367** (2015), pp. 189–202.
